# Supplementary material for: Collaborative intelligence in AI: Evaluating the performance of a council of AIs on the USMLE
Source: PLOS Digit Health. 2025 Oct 9;4(10):e0000787. doi: 10.1371/journal.pdig.0000787 (PMC12510544; doi:10.1371/journal.pdig.0000787)
Supplement: S2 File — (ZIP) [file pdig.0000787.s002.zip › S2 Data Raw data files containing unprocessed question inputs and LLM outputs including transcripts of deliberations/STEP3_Question-35-1_Type-MC-NJ.docx]

**Facilitator** **[Round 1]:** A 26-year-old woman comes to the office 1 day after she passed bloody mucus with her stool. She noticed mild abdominal cramping and some constipation preceding the episode; she has not had diarrhea or melanotic stools. She has not had any exposure to unusual or poorly kept food or to any known infectious agents. She says she has been under considerable stress because she is anticipating the defense phase of her PhD thesis soon. Her only medication is acetaminophen for occasional headaches. Her father had colon cancer and died at age 48 years from metastatic disease. She recalls being told that an aunt died of an unknown abdominal tumor. The patient's temperature is 37.6°C (99.6°F), pulse is 90/min, respirations are 18/min, and blood pressure is 110/74 mm Hg. Abdomen is mildly distended, nontympanitic, and tender to palpation only in the right lower quadrant. There is no rebound tenderness or guarding. Pelvic examination shows no masses or mucosal lesions. Rectal examination elicits pain and bloody mucus. Which of the following is the most appropriate diagnostic study? (A) Colonoscopy (B) Esophagogastroduodenoscopy (C) Lower gastrointestinal barium study (D) Ultrasonography of the appendix (E) Upper gastrointestinal barium study with small-bowel follow-through

**AI_D:** RESTATING THE QUESTION: The patient, a 26-year-old woman, is experiencing some worrying gastric health symptoms like passing bloody mucus with her stool, mild abdominal cramping, and constipation, along with absence of infectious agents or unusual dietary exposure. She doesn’t have diarrhea or melanotic stools. She is under significant stress and her only known medication is acetaminophen for headaches. Her family has a history of abdominal tumors and colon cancer. Apart from a slightly distended abdomen and some tenderness, her physical check-up was largely normal. We are looking for the most appropriate diagnostic study to further investigate her condition. PLANNING: The critical assumption is that she might have a condition related to her family history of colon cancer, such as a malignant colorectal condition. Another assumption could be that she has an unrelated gastric issue causing the bloody mucus, like an infection, ulceration or inflammation. But since there are no signs of infectious agents, this becomes less likely. After generating and evaluating all assumptions, we hypothesize that a condition related to her family history of colon cancer may be clinically prioritized for further investigation. CLINICAL REASONING: Looking at her symptoms, including bloody mucus and abdominal cramping, along with the fact that her father had colon cancer, it prompts one to consider a colorectal issue. The absence of signs of infectious agents, diarrhea, or change in dietary exposure point away from gastrointestinal infectious disease. Her elevated stress levels can cause exacerbation of symptoms, but are not likely the root cause. A colorectal cancer presents with changes in bowel habits and blood in the stool, often without pain, as seems to be the case here. Given her family history and current symptoms, a diagnostic tool focusing on the colon would be best. CONCLUDING: Following my clinical reasoning, I anticipate that the most appropriate diagnostic study in this case would enable us to examine her colon as that is where the symptoms seem to point and where her family history lies. RESPONSE: Consequently, the most fitting diagnostic study, given all the information and considerations, would be (A) Colonoscopy. This would allow for direct visualization and biopsy of any suspicious lesions found in the colon, and potentially early detection and treatment of a serious condition like colorectal cancer.

**AI_B:** (1) RESTATING THE QUESTION: The question presents the scenario of a 26-year-old woman who is suffering from blood stained mucus in her stool along with mild abdominal pain and constipation. She has a family history of colorectal cancer, and recently she has been experiencing a high level of stress. She is not aware of exposure to any known infectious agents and her diet has not involved any suspicious food. Her only medication is acetaminophen which she uses sparingly for her headaches. Her physical exam shows abdominal tenderness in the right lower quadrant and pain in rectal examination. We are asked to identify the most suitable diagnostic test. (2) PLANNING: The patient's symptoms may suggest symptoms of lower gastrointestinal tract pathology such as diverticulitis or inflammatory bowel diseases but given her family history, colorectal cancer should be ruled out as well. When assessing the diagnostic choices, colonoscopy (A) and lower gastrointestinal barium study (C) both cater to the lower gastrointestinal tract. Esophagogastroduodenoscopy (B), ultrasonography of the appendix (D), and upper gastrointestinal barium study (E) govern the upper gastrointestinal, appendix, and upper gastrointestinal and small bowel, respectively. (3) CLINICAL REASONING: The symptoms direct towards a pathology in the lower gastrointestinal tract. The right lower quadrant pain could be caused by appendicitis, but the absence of rebound tenderness or guarding makes this less likely. Moreover, the symptoms, notably passing of blood and mucus and constipation, doesn't align with esophageal/gastric or small bowel pathology that esophagogastroduodenoscopy (B) or upper GI barium study (E) can confirm or rule out. The significant family history of colorectal cancer are key aspects to this patient's presentation which cannot be ignored. A colonoscopy would give a comprehensive view of the entire colon and rectum and is more suited than a lower GI barium study (C), as it would allow for visual inspection of all regions and potential biopsy for further examination. (4) CONCLUDING: In the context of bloody stools, right lower quadrant abdominal pain, family history of colorectal cancer, and normal vital signs, a condition in the lower GI tract is highly probable. In such a context, a colonoscopy would be the most suitable method to visualize the entire colorectal mucosa and to biopsy if needed. (5) RESPONSE: The most appropriate diagnostic study for this patient would be (A) Colonoscopy.

**AI_C:** (1) RESTATING THE QUESTION: The question is this: Given this young woman's medical history and symptoms, which of the listed diagnostic studies is most appropriate to administer? She has recently passed bloody mucus with her stool and experienced abdominal cramping and constipation. She is stressed and takes only acetaminophen. Her family has a history of abdominal cancer. Her physical examination was mostly unremarkable, except for distention and mild tenderness of the abdomen at the right lower quadrant and the presence of bloody mucus on rectal examination. (2) PLANNING: One assumption in this case is that her symptoms are due to non-cancerous causes, such as diet, stress, or gastrointestinal infections. However, given her family history of colon cancer and unknown abdominal tumors, another assumption to generate is that these symptoms could potentially be indicators of early-stage colorectal cancer. The evaluation and prioritization of these assumptions should take into account the patient's familial history and the presence of blood in her stool, indicating a more serious problem that would need to be addressed promptly. A focused approach to rule out potential colorectal cancer would be most clinically prudent. (3) CLINICAL REASONING: In evaluating these options, we need to consider which diagnostic study has the potential to provide the most accurate clinical picture given her symptoms. Although stress and poor diet could lead to constipation and other gastrointestinal disturbances, the presence of bloody mucus in her stool is concerning for a more serious issue, such as a polyp or cancer, particularly given her family history. Having a colonoscopy (option A) would allow direct visualization and biopsy of any suspicious lesions that might give rise to these symptoms. It would also help in ruling out a potential hereditary colon cancer syndrome linked with her family history. (4) CONCLUDING: Esophagogastroduodenoscopy, lower GI barium study, ultrasound of the appendix, and upper GI barium study with small-bowel follow-through (options B, C, D, E) can be useful in certain conditions, but given the specifics of the patient’s presentation and her family history, they are less likely to provide as much direct, applicable information compared to a colonoscopy. (5) RESPONSE: Thus, the most appropriate diagnostic study given this patient's familial history and presentation would be a (A) Colonoscopy.

**AI_A:** (1) RESTATING THE QUESTION: A 26-year-old woman presents with rectal bleeding, abdominal discomfort, and constipation. She does not report any high-risk exposures, notable dietary changes, or digestive symptoms such as diarrhea or melanotic stools. She is currently under high stress and only takes acetaminophen for occasional headaches. Her family history includes her father who died of colon cancer and an aunt who died of an unspecified abdominal tumor. Physical examination reveals mild abdominal distention and right lower quadrant tenderness. Pelvic examination is unremarkable. The woman's rectal examination engenders discomfort and reveals bloody mucus. We need to decide which diagnostic procedure would yield the most relevant and reliable information for her case. (2) PLANNING: Assumptions include that her symptoms are the result of an underlying gastrointestinal (GI) disease process or disorder, possibly associated with her family history of abdominal cancers. Alternate assumptions could include that this is an isolated episode related to stress and acetaminophen use, or symptoms could be related to gynecological conditions, given the right lower quadrant tenderness. The clinically prioritized assumption would be to consider her symptoms as potential indicators of an underlying GI disorder, especially due to her family history of colon cancer. (3) CLINICAL REASONING: Let's connect these dots. The presence of bloody mucus suggests a GI origin, likely from the lower GI tract considering her symptoms (abdominal discomfort, constipation, and blood in stool). This, combined with her positive family history of abdominal cancers (particularly colon cancer), can guide us to prioritize investigations focusing on the lower GI system. Esophagogastroduodenoscopy (choice B) and upper GI barium study with small-bowel follow-through (choice E) primarily investigate the upper GI tract, which does not correlate with her primary symptoms. Ultrasonography of the appendix (choice D) is typically reserved for acute appendicitis presentations, which she does not demonstrate. Lower GI barium study (choice C) can provide valuable information but is generally considered less sensitive and specific than a colonoscopy for diagnosing colon pathologies, including colorectal cancers. (4) CONCLUDING: Thus, using clinical reasoning based on her symptoms, physical examinations, and family history, a test that can examine her whole colon thoroughly would be the most favorable diagnostic study to proceed with. (5) RESPONSE: Therefore, the most appropriate diagnostic study for this patient would be a Colonoscopy (Choice A).

**AI_E:** RESTATING THE QUESTION: A 26-year-old woman presents with symptoms suggesting an issue in her lower abdominal or gastrointestinal system, specifically right lower quadrant pain, bloody mucus in stool, abdominal cramping, and constipation without any known and specific dietary or infectious cause. Her past medical history is significant for family history of colon cancer in her father and a possible abdominal tumor in her aunt. We are tasked to make a decision on the most suitable diagnostic study to further reach the correct diagnosis. PLANNING: Our assumptions here are based on the clinical signs and symptoms, as well as the family's cancer history. This might suggest a problem in the lower end of her gastrointestinal tract, possibly related to colon or rectal issues that could be benign, pre-malignant, or malignant. Other possibilities include an issue with her appendix or other parts of her gastrointestinal tract. To verify these assumptions, we have to consider the following diagnostic options: colonoscopy, esophagogastroduodenoscopy, lower gastrointestinal barium study, ultrasonography of the appendix, and an upper gastrointestinal barium study with small-bowel follow-through. CLINICAL REASONING: The presence of bloody mucus in the stool and tenderness in the right lower quadrant may suggest disease in the colon or rectum, such as colorectal cancer, diverticulitis, or inflammatory bowel disease. Given the strong family history of colon cancer, the likelihood of having a colorectal problem is increased. The colon is fully examined with a colonoscopy, including the rectal area where the bloody mucus potentially originates. Other modalities like an esophagogastroduodenoscopy primarily investigate the upper two-thirds of the digestive tract, while an upper gastrointestinal study with small bowel follow-through focuses on the upper digestive tract and small intestine. As these areas are not likely the cause in this patient's case based on the symptoms, these diagnostic modalities may not be as helpful. A lower gastrointestinal barium study could be useful, but it is less sensitive and specific than a colonoscopy for detecting colorectal pathology. Ultrasonography of the appendix might be considered if acute appendicitis is suspected. However, apart from the right lower quadrant tenderness, her other symptoms and family history make this less likely. CONCLUDING: By process of elimination and based on the most likely diagnosis due to her symptoms and significant family history - in a setting of colorectal or possible lower gastrointestinal pathology, colonoscopy is the most suited diagnostic modality as it can directly visualize the inner surface of the colon where we suspect the problem is likely to be located. RESPONSE: So, in regard to this 26-year-old woman's symptoms and her family history of colon cancer, the most appropriate diagnostic study would be (A) Colonoscopy.

**Facilitator** The consensus among the AI responses is that the most appropriate diagnostic study for the 26-year-old woman presenting with rectal bleeding, abdominal discomfort, and constipation, and with a family history of colon cancer, would be a Colonoscopy (Choice A). This conclusion is based on the following reasoning: The woman's symptoms, particularly the presence of bloody mucus, suggest a gastrointestinal (GI) origin, likely from the lower GI tract. This, combined with her family history of abdominal cancers, particularly colon cancer, guides the focus towards investigations of the lower GI system. The other diagnostic options, such as Esophagogastroduodenoscopy (Choice B) and Upper GI barium study with small-bowel follow-through (Choice E), primarily investigate the upper GI tract, which does not correlate with her primary symptoms. Ultrasonography of the appendix (Choice D) is typically reserved for acute appendicitis presentations, which she does not demonstrate. Lower GI barium study (Choice C) can provide valuable information but is generally considered less sensitive and specific than a colonoscopy for diagnosing colon pathologies, including colorectal cancers. A colonoscopy would allow for a comprehensive view of the entire colon and rectum, enabling visual inspection of all regions and potential biopsy for further examination. Given the patient's symptoms and family history, a condition in the lower GI tract is highly probable, and a colonoscopy would be the most suitable method to visualize the entire colorectal mucosa and to biopsy if needed. Therefore, the consensus is that the most appropriate diagnostic study for this patient would be a Colonoscopy (Choice A).
